# Lutein Induces Reactive Oxygen Species-Mediated Apoptosis in Gastric Cancer AGS Cells via NADPH Oxidase Activation

**DOI:** 10.3390/molecules28031178

**Published:** 2023-01-25

**Authors:** Ju Won Eom, Joo Weon Lim, Hyeyoung Kim

**Affiliations:** Department of Food and Nutrition, College of Human Ecology, Yonsei University, Seoul 03722, Republic of Korea

**Keywords:** apoptosis, gastric cancer cells, lutein, NF-κB, reactive oxygen species

## Abstract

Disruption of apoptosis leads to cancer cell progression; thus, anticancer agents target apoptosis of cancer cells. Reactive oxygen species (ROS) induce apoptosis by activating caspases and caspase-dependent DNase, leading to DNA fragmentation. ROS increase the expression of apoptotic protein Bax, which is mediated by activation of nuclear factor-κB (NF--κB). Nicotinamide adenine dinucleotide phosphate (NADPH) oxidase is an important source of endogenous ROS, and its activation is involved in apoptosis. Lutein, an oxygenated carotenoid and known antioxidant, is abundant in leafy dark green vegetables, such as spinach and kale, and in yellow-colored foods, such as corn and egg yolk. High amounts of lutein increase ROS levels and exhibit anticancer activity. However, its anticancer mechanism remains unclear. This study aimed to determine whether lutein activates NADPH oxidase to produce ROS and induce apoptosis in gastric cancer AGS cells. Lutein increased ROS levels and promoted the activation of NADPH oxidase by increasing the translocation of NADPH oxidase subunit p47 ^phox^ to the cell membrane. It increased NF-κB activation and apoptotic indices, such as Bax, caspase-3 cleavage, and DNA fragmentation, and decreased Bcl-2, cell viability, and colony formation in AGS cells. The specific NADPH oxidase inhibitor ML171, and the known antioxidant N-acetyl cysteine reversed lutein-induced cell death, DNA fragmentation, and NF-κB DNA-binding activity in AGS cells. These results suggest that lutein-induced ROS production is dependent on NADPH oxidase, which mediates NF-κB activation and apoptosis in gastric cancer AGS cells. Therefore, lutein supplementation may be beneficial for increasing ROS-mediated apoptosis in gastric cancer cells.

## 1. Introduction

Gastric cancer is the fifth most common cancer and was the third most deadly cancer in 2020 [1]. Risk factors leading to gastric cancer include *Helicobacter pylori* (*H. pylori*) infection, unhealthy lifestyles, and genetic predisposition [2]. *H. pylori* infection is considered the most important cause of gastric cancer [3]. Lifestyle, including tobacco smoking, alcohol drinking, a diet high in sodium, and intake of too little fresh vegetables or fruits, may increase the risk of gastric cancer [2,3,4,5,6]. Therefore, *H. pylori* eradication, smoking control, and healthy diet may reduce the risk of gastric cancer.

Apoptosis is a highly regulated process of programmed cell death that is essential for the development and survival of multicellular organisms [7]. Organisms need to discard potentially harmful cells that have accumulated mutations or become infected by pathogens. Apoptosis involves a characteristic set of morphological and biochemical events whereby cells undergo a cascade of self-destruction. Therefore, proper regulation of apoptosis is critical for maintaining normal cellular homeostasis [8]. However, disruption of apoptosis signaling cannot block cancer progression, thus many cytotoxic anticancer agents involve induction of apoptosis [9].

Reactive oxygen species (ROS) are known to induce apoptosis, especially when cells lack antioxidant capacities. Although cancer cells produce ROS, antioxidant enzyme levels are low in most human cancers [10]. Therefore, ROS-producing agents may be beneficial for reducing the survival of cancer cells.

In the initiation and regulation of the apoptotic cascade, ROS trigger the release of cytochrome c from the mitochondria into the cytoplasm, where it induces caspase activation. Initiator caspases activate effector caspases such as caspase-3, through cleavage at a specific site [11]. Activated caspase-3 cleaves various proteins including nuclear enzyme poly (ADP-ribose) polymerase (PARP) [12] and an inhibitor of caspase-activated DNase (ICAD), leading to the release of caspase-activated DNase (CAD) to degrade chromosomal DNA [13]. Thus, DNA fragmentation is an indicator of apoptosis.

Bcl-2 is an anti-apoptotic molecule that promotes cellular survival, while decreased Bcl-2 induces apoptotic cell death [14]. Bax is a pro-apoptotic molecule that translocates from the cytosol to the outer mitochondrial membrane, forming an oligomer that is crucial for mitochondrial membrane permeabilization [15]. Mitochondrial changes, including cytochrome c release and loss of membrane permeabilization, are prevented by Bcl-2 [16].

Therefore, caspase-3 activation and Bax/Bcl-2 levels indicate apoptotic cell death. Disruption of apoptosis signaling cannot block cancer progression; thus, certain anticancer agents are able to induce apoptosis [17].

Nicotinamide adenine dinucleotide phosphate (NADPH) oxidase comprises a group of membrane-associated enzymes that produce superoxide from oxygen and NADPH [18]. This superoxide is rapidly converted into H_2_O_2_ [19]. Thus, NADPH oxidase is an important source of ROS in cells. NADPH oxidase consists of membrane-bound components such as gp91^phox^ and cytosolic components such as p47^phox^ [20]. It is activated when cytosolic components migrate to the plasma membrane and associate with the membrane-bound components. Nox1 is a gp91^phox^ homologue and can bind with the cytosolic component p47^phox^ [21]. Nox1 protein is expressed in human gastric adenocarcinomas but is absent in normal tissues [22,23]. Therefore, targeting NADPH oxidase may be an effective therapeutic approach for inhibiting progression of gastric cancer. We previously showed that NADPH oxidase activation induced by large amounts of astaxanthin induced gastric cancer cell death [24].

Nuclear factor -κB (NF-κB) is a transcriptional factor that regulates a variety of cellular processes including immune response, inflammatory response, proliferation, and apoptosis. Normally, NF-κB is localized in the cytosol and is inactivated upon binding to IκB proteins. Upon stimulation by a certain signal, IκB proteins are phosphorylated by the IκB kinase complex and undergo proteasomal degradation, leading to the activation of NF-κB. ROS activate NF-κB by increasing the phosphorylation and degradation of IκBα [25]. In relation to NF-kB activation and cancer cell death, NF-κB has been reported to play a pro-apoptotic role in cisplatin-induced cell death in head and neck squamous carcinoma cells [26]. NF-κB mediates apoptosis in adenocarcinoma cells infected with retrovirus [27] or treated with TNF-α [28]. In glioblastoma cell lines, NF-κB exerts a pro-apoptotic function in TNF-related apoptosis-inducing ligand (TRAIL)-induced apoptosis, which can be reversed by overexpression of the dominant negative IκBα super-repressor [29]. Taken together, NADPH activation-derived ROS and NF-κB activation may increase apoptotic indices (caspase-3 activation, Bax/Bcl-2 levels, and DNA fragmentation) and cell death in cancer cells.

Lutein, a member of the xanthophyll family of carotenoids, is a yellow-orange pigment commonly found in plants [30]. Dietary sources of lutein include dark leafy green vegetables such as spinach and kale, and yellow-colored foods such as corn and egg yolk [30]. Lutein has anti-inflammatory, anti-oxidant, and anti-cancer properties [31,32]. ROS activates inflammatory signaling and induces development of metabolic and inflammatory diseases. Lutein maintains endogenous antioxidant (glutathione) and activates nuclear factor erythroid 2–related factor 2 (Nrf2) and Nrf2 signaling-mediated expression of antioxidant enzymes (glutathione peroxidase, superoxide dismutase, catalase), thereby reducing ROS levels and inhibiting the expression of inflammatory mediators [33]. Therefore, lutein supplementation may prevent oxidative stress-induced inflammatory disorders including neurodegenerative disorders, diabetic retinopathy, and cardiovascular diseases.

Epidemiological studies have shown that dietary intake of lutein decreases the risk for breast and gastric cancers [34,35,36]. Lutein exerts toxic and inhibitory effects on several cancer cell lines including cervical, prostate, breast, and hepatic cancer cells [37,38,39,40]. In terms of its anticancer mechanism, lutein increased intracellular ROS levels and inhibited cell growth via cell-cycle arrest in breast cancer cells [41], and induced apoptosis in human cervical cancer HeLa cells by enhancing ROS production [42]. Therefore, it is necessary to determine whether lutein increases ROS by activating NADPH oxidase thereby inducing apoptosis in gastric cancer cells.

The current study aimed to investigate whether lutein induces apoptosis through NADPH oxidase-mediated ROS production and subsequent NF-κB activation in gastric cancer AGS cells, by evaluating cell viability, colony formation, and apoptotic indices (Bax, Bcl-2, caspase-3 activation, and DNA fragmentation). The involvement of NADPH oxidase and ROS in apoptosis and NF-κB activation in AGS cells was assessed by treating the cells with a specific inhibitor of NADPH oxidase, ML171, and a known antioxidant, N-acetyl cysteine (NAC), along with lutein treatment.

To determine the effect of lutein on normal gastric epithelial RGM cells, cell viability was measured in RGM cells treated with lutein. In addition, to determine the effect of lutein on other human gastric cancer cells, including MKN-74, MKN-1, and SNU-668, ROS levels and cell viability were assessed in cells treated with lutein. To investigate whether lutein sensitizes cancer cells to the chemotherapeutic drug etoposide, the cells (AGS, MKN-74, MKN-1, and SNU-668, as well as RGM) were treated with etoposide with or without low concentrations of lutein, and cell viability was determined.

## 2. Results

### 2.1. Lutein Increases Apoptotic Indices in AGS Cells, but Does Not Affect Cell Viability in RGM Cells

**Figure 1:**
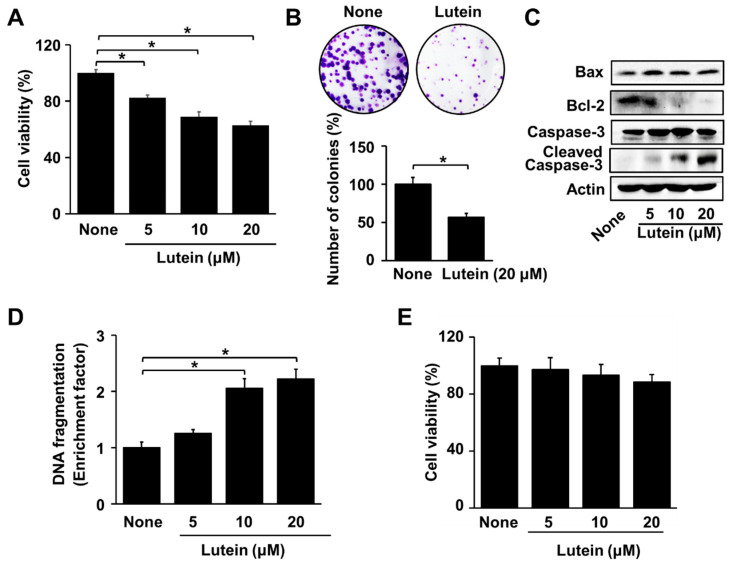
Effects of lutein on cell viability, colony formation, Bax and Bcl-2 levels, caspase-3 cleavage, and DNA fragmentation in AGS cells, and cell viability in RGM cells (**A**) AGS cells were treated with the indicated concentrations of lutein for 24 h. Cell viability was determined by counting the number of viable cells. The viability of untreated cells (none) was set at 100%. (**B**) AGS cells were treated with 20 μM lutein and incubated for 2 weeks, after which the colonies were visualized by staining with crystal violet. The number of cell clones were counted using a microscope. The number of untreated cell clones (indicated as “none”) was set as 100%. (**C**) AGS cells were treated with the indicated concentrations of lutein for 24 h. The levels of Bax, Bcl-2, caspase-3, and cleaved caspase-3 in whole-cell extracts were determined by Western blotting. (**D**) AGS cells were treated with the indicated concentrations of lutein for 20 h. DNA fragmentation was assessed by the amount of nucleosome-bound DNA in the cell lysates. The level of DNA fragmentation for the untreated cells (“none”) was set as 1. * *p* < 0.05. (**E**) RGM cells were treated with the indicated concentrations of lutein for 24 h. Cell viability was determined by counting the number of viable cells. The viability of untreated cells (“none”) was set at 100%. * *p* < 0.05.

Treatment with lutein (at 5, 10, and 20 μM) for 24 h decreased cell viability in a dose-dependent manner (Figure 1A). At 20 μM of lutein, viable cell numbers were approximately 50% of untreated cells. Thus, for the colony formation assay, the cells were treated with 20 μM of lutein. As shown in Figure 1B, two weeks of treatment with lutein (at 20 μM) reduced the number of colonies, indicating that lutein decreased the number of viable cells. At 24 h of culture, lutein (at 5, 10, and 20 μM) increased the protein levels of Bax and cleaved caspase-3, but decreased the protein levels of Bcl-2 in a dose-dependent manner (Figure 1C). Similar to the results for cell death (Figure 1A), 20 μM of lutein showed significant effects on Bcl-2 loss, Bax induction, and caspase-3 cleavage in AGS cells. Lutein (at 5, 10, and 20 μM) increased DNA fragmentation dose-dependently, as measured by the amount of oligonucleosome-bound DNA in the cell lysate (Figure 1D). Lutein at a dose of 20 μM induced higher levels of DNA fragmentation compared with 5 and 10 uM doses. Based on these results, 20 µM concentration of lutein was selected for further experiments on NADPH oxidase activation, ROS production, NF-kB activation, and apoptosis. However, lutein (at 5, 10, and 20 μM) had no effect on the viability of normal gastric RGM cells (Figure 1E).

### 2.2. Lutein Sentitizes AGS Cells to Etoposide for Cell Death

**Figure 2:**
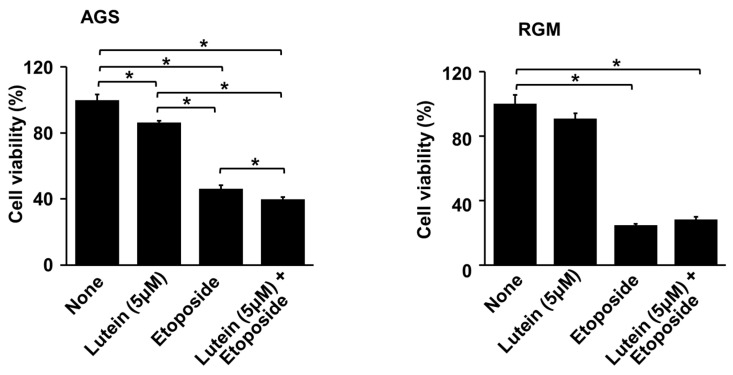
Effect of lutein on etoposide-induced cell death in AGS cells and RGM cells. The cells were pre-treated with 5 μM lutein for 4 h and then treated with 10 μM etoposide for 24 h. Cell viability was determined by counting the number of viable cells. The viability of untreated cells (“none”) was set at 100%. * *p* < 0.05.

To determine whether lutein sensitizes AGS cells to the anticancer agent etoposide, the cells were treated with etoposide (10 μM) with or without relatively a low concentration of lutein (5 μM), and cell viability was determined. Because etoposide inhibits DNA synthesis by forming a complex with topoisomerase II and DNA and induces apoptosis [43,44], it decreased cell viability in AGS cells and RGM cells (Figure 2). The combination treatment of lutein (5 µM) and etoposide significantly suppressed the viability of AGS cells. Cell death induced in AGS cells by etoposide and lutein in combination was higher than that induced by lutein alone or etoposide alone (Figure 2, left panel). However, lutein had no synergetic effect on etoposide-induced cell death in RGM cells (Figure 2, right panel).

### 2.3. Lutein Increases ROS Levels and NADPH Oxidase Activity in AGS Cells

**Figure 3:**
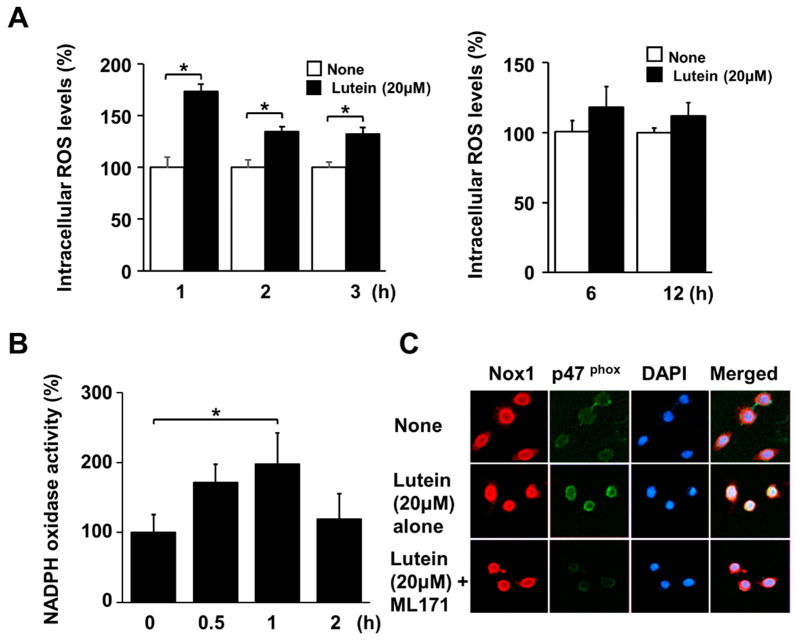
Effects of lutein on intracellular ROS levels, NADPH oxidase activity, and translocation of p47^phox^ in AGS cells. (**A**,**B**) Cells were treated with 20 μM lutein for the indicated time periods. (**A**) Intracellular ROS levels were measured by DCFH-DA assay. The intracellular ROS levels of untreated cells (“none”) were set at 100%. * p < 0.05 (**B**) NADPH oxidase activity in the membrane fraction was measured using lucigenin assay. The NADPH oxidase activity at 0 h was set at 100%. (**C**) The cells were pre-treated with 20 μM ML171 for 1 h and then treated with 20 μM lutein for 1 h. Immunocytochemistry of AGS cells was carried out to detect Nox1 and p47 ^phox^. Nox1 and p47^phox^ were visualized using fluorescein/rhodamine-conjugated anti-rabbit IgG (red) and FITC-conjugated anti-mouse IgG (green) with DAPI counterstaining (blue) of the same field, respectively.

To determine the effect of lutein on ROS production, intracellular ROS levels were measured by DCFH-DA assay. Lutein (20 μM) increased ROS levels at 1 h, and continued to do so until 3 h (Figure 3A, left panel). However, lutein did not increase ROS levels at 6 h or 12 h (Figure 3A, right panel). Lutein enhanced NADPH oxidase activity in AGS cells at 1 h, which decreased at 2 h (Figure 3B). These results show that lutein increased NADPH oxidase activity and ROS levels in AGS cells at 1h, continuing to do so until 3 h.

To examine how lutein activated NADPH oxidase, immunocytochemistry was carried out. Cells were treated with 20 μM lutein with or without ML171 for 1 h, and the levels of Nox1 and p47^phox^ were determined. Nox1 was detected using tetramethyl rhodamine isothiocyanate (TRITC), which emits red fluorescence, while p47^phox^ was detected using fluorescein-5-isothiocyanate (FITC), which emits green fluorescence. When the two different fluorescence signals merged, they appeared yellow. Lutein treatment increased the translocation of NADPH oxidase subunit p47^phox^ from the cytosol to the plasma membrane (Figure 3C, second row). The Nox inhibitor ML171 prevented lutein-induced translocation of p47^phox^ in AGS cells (Figure 3C, 3rd lane). However, the membrane subunit of NADPH oxidase Nox1 did not change upon lutein treatment (Figure 3C, first column).

### 2.4. ML171 and NAC Inhibit Lutein-Induced Increase in ROS and Cell Death in AGS Cells

**Figure 4:**
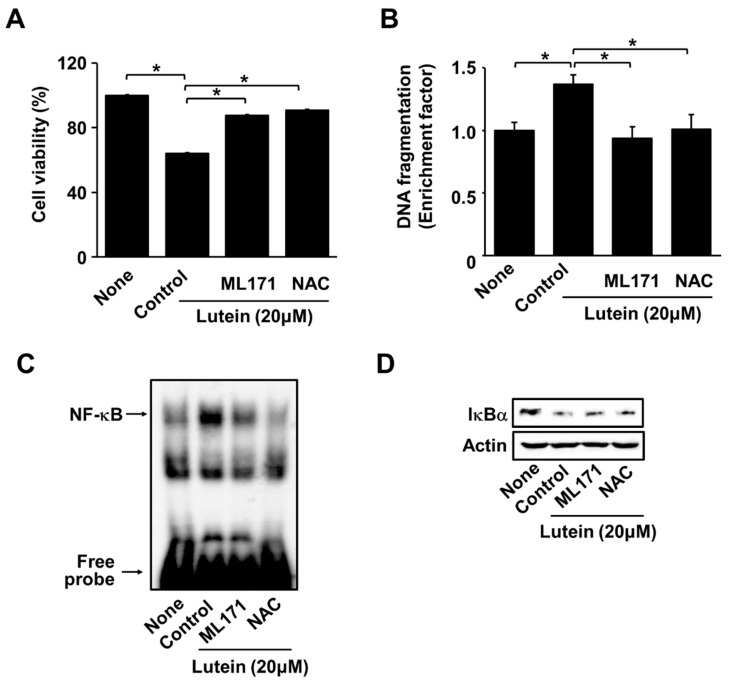
Effect of ML171 and NAC on cell viability, DNA fragmentation, NF-κB-DNA binding activity in AGS cells treated with lutein. Cells were pre-treated with ML171 or NAC for 1 h and then treated with 20 μM of lutein and incubated for (**A**) 24 h, (**B**) 20 h or (**C**,**D**) 1 h. (**A**) Cell viability was determined by counting the number of viable cells. Cel viability of untreated cells (“none”) was set at 100%. * *p* < 0.05; (**B**) DNA fragmentation was assessed by the amount of nucleosome-bound DNA in the cell lysates. The level of DNA fragmentation of untreated cells (“none”) was set as 1. * *p* < 0.05; (**C**) The DNA-binding activity of NF-κB was examined by EMSA. (**D**) IκBα levels in whole-cell lysate were determined by Western blot analysis, using actin as loading control.

To examine whether the reduction in cell viability was related to the activation of NADPH oxidase and the pro-oxidant properties of lutein, cells were treated with an NADPH oxidase inhibitor (ML171) or an antioxidant NAC before lutein treatment. Pretreatment with ML171 or NAC suppressed lutein-induced reduction in cell viability (Figure 4A). Similarly, ML171 and NAC prevented lutein-induced DNA fragmentation (Figure 4B). These results suggest that lutein induces cell death via NADPH oxidase-mediated ROS production in AGS cells.

As shown in Figure 4C,D, ML171 and NAC inhibited lutein-induced increases in NF-κB-DNA binding activity and decreases in IκBα in AGS cells. These results indicate that lutein-induced activation of NF-κB is mediated by NADPH oxidase and ROS in AGS cells.

### 2.5. Lutein Decreases Cell Viability, but Increases ROS Levels in Human Gastric Cancer MKN-74, MKN-1 and SNU-668 Cells

**Figure 5:**
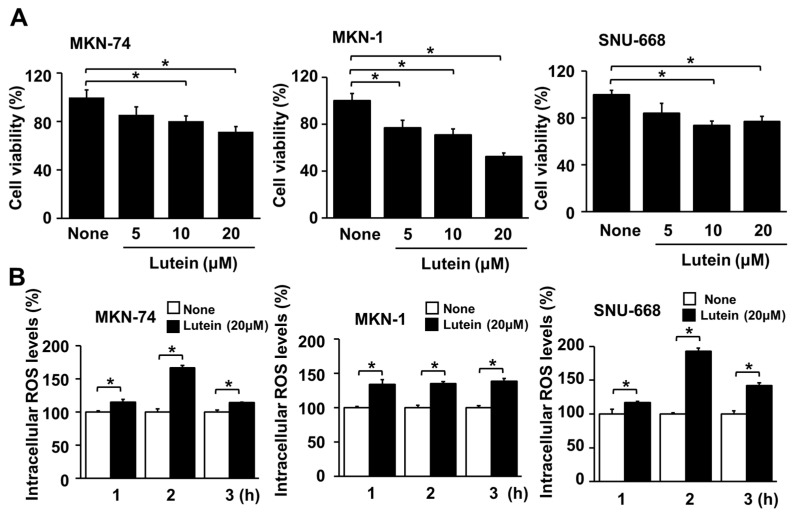
Effect of lutein on cell viability and intracellular ROS levels in MKN-74, MKN-1, and SNU-668 cells. (**A**) MKN-74, MKN-1, and SNU-668 cells were treated with the indicated concentrations of lutein for 24 h. Cell viability was determined by counting the number of viable cells. The viability of cells treated without lutein (“none”) was set at 100%. * p < 0.05; (**B**) MKN-74, MKN-1, and SNU-668 cells were treated with 20 μM lutein for the indicated time periods. Intracellular ROS levels were measured by DCFH-DA assay. The intracellular ROS levels of untreated cells (“none”) were set at 100%. * p < 0.05.

As shown in Figure 5A, 24 h treatment with lutein (10 and 20 μM) significantly reduced cell viability in MKN-74, MKN-1, and SNU-668 cells. Lutein (20 μM) increased ROS levels in MKN-74, MKN-1, and SNU-668 cells at 1 h, and the increase continued up to 3 h (Figure 5B). These results show that lutein-induced cell death is related to ROS production in MKN-74, MKN-1, and SNU-668 cells.

### 2.6. Lutein (5 μM) Increases Etiposide-Induced Cell Death in MKN-1 Cells

**Figure 6:**
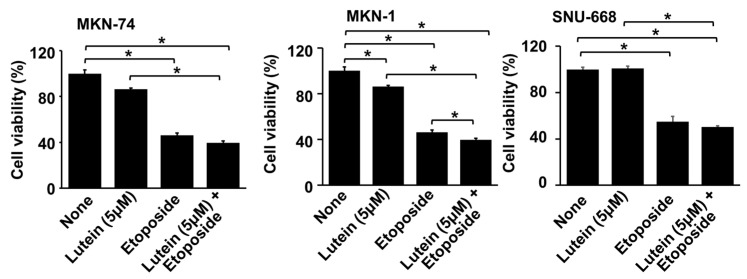
Effects of lutein on etoposide-induced cell death in MKN-74, MKN-1, and SNU-668 cells. The cells were pre-treated with 5 μM lutein for 4 h and then treated with 10 μM etoposide for 24 h. Cell viability was determined by counting the number of viable cells. The viability of untreated cells (“none”) was set at 100%. * *p* < 0.05.

To determine whether lutein sensitizes MKN-74, MKN-1, and SNU-668 cells to etoposide, the cells were treated with etoposide (10 μM) with or without lutein (5 μM), and cell viability was determined. Etoposide decreased cell viability in MKN-74, MKN-1, and SNU-669 cells (Figure 6). Lutein (5 μM) decreased cell viability in MKN-1 cells, while this low concentration of lutein did not affect cell viability in MKN-74 and SNU-669 cells. Combined treatment with etoposide and lutein caused a synergistic reduction of cell viability in MKN-1 cells, compared with treatment by lutein alone or etoposide alone. Because etoposide significantly induces cell death, a relatively low dose of lutein was used to determine the synergistic effect of lutein on etoposide-induced cell death in the present study.

## 3. Discussion

**Figure 7:**
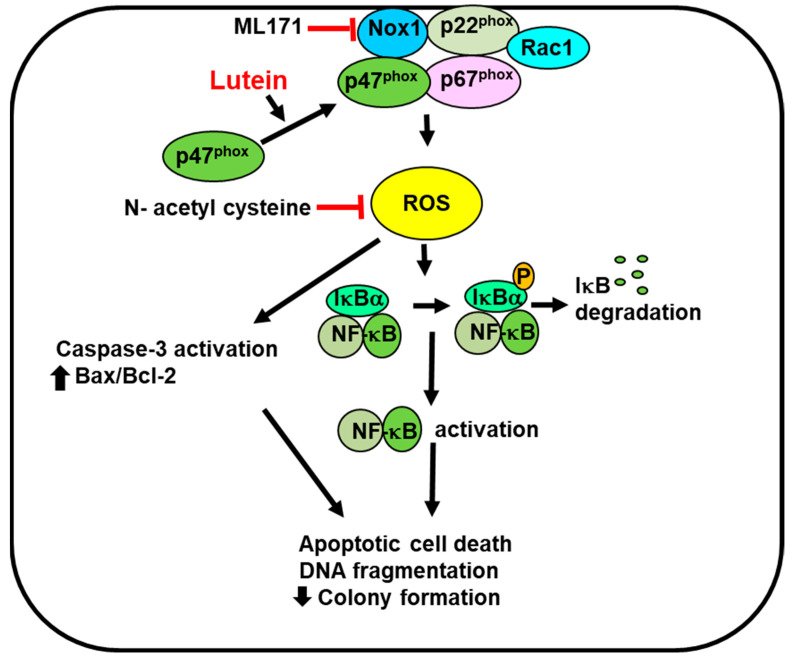
The proposed mechanism through which lutein stimulates NADPH oxidase, production of reactive oxygen species, NF-κB activation, and apoptosis in gastric cancer AGS cells. Lutein increases NADPH oxidase activity by stimulating the translocation of cytosolic NADPH oxidase subunit p47 ^phox^ to the cell membrane to form a complex with membrane-bound subunit Nox1. It increases reactive oxygen species (ROS) production that induces degradation of IκBα and activates NF-κB. ROS stimulate caspase-3 cleavage and increase the Bax/BCl-2 ratio. These apoptosis signals induce DNA fragmentation, decreased colony formation, and promote apoptotic cell death. The specific NADPH oxidase inhibitor ML171 and the known antioxidant N-acetyl cysteine inhibit lutein-induced increases in ROS levels, NF-κB activation, DNA fragmentation, and apoptotic cell death in gastric cancer AGS cells. The results demonstrate that lutein-induced ROS production is dependent on NADPH oxidase, which mediates NF-κB activation and apoptosis in gastric cancer cells. Therefore, lutein supplementation may be beneficial for increasing ROS-mediated gastric cancer cell apoptosis. NF-κB, nuclear factor -κB; ROS, reactive oxygen species. Red block indicates inhibition.

As summarized in Figure 7, the present study shows that lutein stiimulates NADPH oxidase activity by increasing the translocation of NADPH oxidase subunit p47^phox^ to the cell membrane, to form a complex with membrane-bound subunit Nox1. In AGS cells it increased ROS levels, NF-κB activation, and apoptotic indices such as Bax, caspase-3 cleavage, and DNA fragmentation, and decreased Bcl-2, cell viability, and colony formation. The specific NADPH oxidase inhibitor ML171 and the known antioxidant NAC reduced ROS levels and inhibited lutein-induced increases in ROS levels, NF-κB activation, DNA fragmentation, and apoptotic cell death in gastric cancer AGS cells. The results demonstrate that lutein-induced ROS production is dependent on NADPH oxidase, which mediates NF-κB activation and apoptosis in these gastric cancer cells. Similarly, lutein increased ROS levels and decreased cell viability in MKN-74, MKN-1, and SNU-668 cells. Therefore, lutein supplementation may be beneficial for stimulating ROS-mediated gastric cancer cell death.

Apoptosis has drawn attention due to its significance in cancer therapy. Bcl-2, Bcl-x_L,_ and Bcl-w prevent apoptosis initiation in cells, while Bax responds to cytotoxic signals and induces apoptosis. Caspase-3 is the key executioner of apoptosis, cleaving proteins that are critical for cell survival such as the nuclear enzyme PARP [12,14].

In the present study, lutein increased caspase-3 activity and Bax levels, decreased Bcl-2 levels, and increased ROS levels in AGS cells. The results of the present study are supported by a previous study conducted by Gansukh et al. [42], which showed that lutein derived from marigold (*Tagetes erecta*) petals triggered ROS generation and activated Bax and caspase-3-mediated apoptosis in human cervical carcinoma (HeLa) cells. However, it did not clarify the mechanism by which lutein increases ROS production in HeLa cells. In the present study, we have shown for the first time that lutein treatment increased the translocation of NADPH oxidase subunit p47^phox^ from the cytosol to the plasma membrane to form a complex with the membrane-bound subunit of NADPH oxidase Nox1. Thus, lutein activates NADPH oxidase and produces ROS to activate NF-κB in gastric cancer cells.

High levels of oxidative stress are detrimental to cells, whereas low levels induce their proliferation, indicating that cellular responses to oxidative stress might be different. One important endogenous source of ROS in tumorigenesis is NADPH oxidase [45]. As mentioned previously, the NADPH oxidase subunit Nox1 is expressed in human gastric cancer cells but not in normal gastric cells [22,23]. Human gastric cells express the NADPH oxidase subunit p47^phox^ [46], and Nox1 interacts with p47^phox^ to activate NADPH oxidase upon induction via various stimuli [21]. To activate NADPH oxidase, p47^phox^ is phosphorylated by certain protein kinases such as mitogen-activated kinases (MAPKs), then translocates to the plasma membrane to form an active complex with a membrane-bound subunit such as Nox1 [47].

A previous study showed that p47^phox^ was phosphorylated by both p38 and extracellular signal-regulated kinase (ERK) in human neutrophils [48]. Lutein treatment promotes ERK phosphorylation in murine macrophages and BV-2 microglia [49,50]. In addition, suppression of ERK activation due to hyperglycemia was rescued by lutein pre-treatment in human retinal pigment epithelial cells [51].

In this study, lutein significantly increased intracellular ROS levels and NADPH oxidase activity simultaneously. The activation of NADPH oxidase upon lutein treatment may be explained by its possible involvement in the phosphorylation of MAPK, such as ERK, in AGS cells. Further studies should be performed to determine whether lutein activates MAPK in AGS cells, thereby resulting in NADPH oxidase activation.

NF-κB activation mediates apoptotic signals during chemotherapy in sarcoma cells [26], TNF-α -treated adenocarcinoma cells [28], and glioblastoma cells [29]. As ROS are known to activate the NF-κB pathway [25], we investigated whether lutein-induced activation of NADPH oxidase mediates NF-κB activation and apoptosis in AGS cells.

To confirm whether NADPH oxidase is involved in apoptosis, we used ML171, a Nox1 specific inhibitor. Since Nox1 expression has been shown to be significantly higher in colon and stomach cancers [23], and non-specific inhibitors such as diphenylene iodonium have issues of specificity and toxicity [52], we chose ML171 to determine whether apoptosis of AGS cells was blocked upon inhibition of NADPH oxidase. The present study showed that cell death induced by a high dose of lutein was reversed by ML171, which inhibited lutein-induced activation of NADPH oxidase in AGS cells. Furthermore, lutein-induced DNA fragmentation and NF-κB activation were reversed upon ML171 treatment.

NAC acts as a powerful antioxidant by stimulating glutathione biosynthesis and directly scavenging free radicals [53]. In the present study, NAC blocked the effects of lutein on cell viability, DNA fragmentation, and NF-κB activation in AGS cells. These results demonstrate that the effect of lutein on apoptosis might be mediated by NADPH oxidase-induced ROS production in AGS cells.

Regarding ROS and cancer cell death, cancer cells produce relatively high levels of ROS since they exhibit increased metabolic rate and hypoxia. Hypoxia is a non-physiological phenomenon which is apparent in many cancer cells. Hypoxia induces increased ROS production [54,55]. High levels of ROS stimulate cancer cell growth [56]. However, excessive amounts of ROS result in cancer cell apoptosis [8]. Cancer cells are susceptible to oxidative stress [57,58], resulting in oxidative stress-induced cancer cell death. It has been suggested that polyphenols, which increased ROS production, can be used as therapeutic agents to induce cancer cell death [59]. Several phytochemicals, non-nutritive chemicals found in plants and food, possess antioxidant capacities at low doses, but high doses induce pro-oxidant activity that leads to cancer cell death [60]. These studies support the present findings which showed that a high dose of lutein (20 μM) induced pro-oxidant activity, leading to apoptosis of gastric cancer cells.

In the present study, we mainly used gastric cancer AGS cells to determine the stimulatory effects of lutein on NADPH oxidase activity, ROS production, NF-kB activation, and apoptosis. Additionally, we used gastric cancer MKN-74, MKN-1, and SNU-669 cells to determine the effects of lutein on ROS levels and cell viaility. The results showed that lutein (20 μM) incerased ROS levels and induced cell death in these gastric cancer cells. To assess whether a low concentration of lutein (5 μM) sensitized gastric cells to etoposide, gastric cancer cells (AGS, MKN-74, MKN-1, SNU-668) and normal gastric mucosal RGM cells were treated with etoposide with or without lutein (5 μM). We found that etoposide induced cell death in gastric cancer cells and RGM cells. Lutein (5 μM) increased etoposide-induced cell death in AGS cells and MKN-1 cells. Further study is necessary to determine whether high doses of lutein can augment etoposide-induced apoptosis in various kinds of cancer cells.

Regarding etoposide and NADPH oxidase activity, etoposide did not affect NADPH oxidase activity in human leukemia, but induced cell death due to inhibition of DNA synthesis [61]. Recent study showed that GLX351322, a NADPH oxidase 4 (NOX4)-derived ROS inhibitor, has an inhibitory effect on thyroid carcinoma cell growth and disrupts the resistance of cancer cells to etoposide [62]. However, the present study demonstrates that lutein increases NADPH oxidase activity and ROS production, augmenting etoposide-mediated cell death in AGS cells. The discrepancy between these studies may be caused by different cell types, or by endogenous and exogenous factors. Further study should be carried out with various types of cancer cells to determine the effect of oxidative stress on chemotherapeutic drugs.

A limitation of this study was that four gastric cancer cell lines and one anticancer agent, etoposide, were employed to determine the effect of lutein on gastric cancer cell apoptosis and to assess whether lutein sensitizes chemotherapeutic drugs for cancer cell death. More cancer cell lines and various kinds of anticancer drugs should be investigated to determine the anticancer activity and mechanism of lutein.

In conclusion, lutein triggers apoptosis by increasing NADPH oxidase activity and ROS levels, thereby inducing NF-κB activation and apoptotic indices in gastric cancer AGS cells. A low dose of lutein augments etoposide-induced cell death in AGS cells. Therefore, supplementation with high amounts of lutein may be beneficial for stimulating ROS-mediated gastric cancer cell apoptosis.

## 4. Materials and Methods

### 4.1. Reagents

Lutein, purchased from Cayman Chemical Co. (Catalog No. 10010811, Ann Arbor, MI, USA), was dissolved in dimethyl sulfoxide (DMSO) according to the product information (Cayman Chemical Co, Ann Arbor, MI, USA), aliquoted, and stored at −80 °C. Final concentrations of 5, 10, or 20 μM were applied to the cells. NADPH oxidase 1 inhibitor ML171 and etoposide were purchased from Sigma-Aldrich (St. Louis, MO, USA) and dissolved in DMSO. Antioxidant NAC (Sigma-Aldrich, St. Louis, MO, USA) was dissolved in distilled water. For each experiment, cells incubated with DMSO alone (less than 0.3%) served as the control.

### 4.2. Cell Lines and Culture Conditions

The human gastric cancer cell line AGS (ATCC CRL-1739; gastric adenocarcinoma) was purchased from the American Type Culture Collection (Manassas, VA, USA). Human gastric cancer cell line MKN-74 was obtained from RIKEN BioResource Center (Tsukuba, Ibaraki, Japan). Human gastric cancer cell lines MKN-1 and SNU-668 were obtained from the Korean Cell Line Bank (KCLB, Seoul, Korea). These cells were cultured in RPMI-1640 medium supplemented with 10% fetal bovine serum (GIBCO-BRL, Grand Island, NY, USA) and antibiotics (100 U/mL penicillin and 100 μg/mL streptomycin). The cells were cultured at 37 °C in a humidified atmosphere containing 95% air and 5% CO_2_.

The normal rat gastric epithelial cell line RGM-1 was obtained from RIKEN BioResource Center (Tsukuba, Ibaraki, Japan). RGM-1 cells were grown in a 1:1 mixture of Dulbecco’s modified Eagle medium (DMEM; GIBCO-BRL, Scotland, UK) and Ham’s F-12 medium (GIBCO-BRL), supplemented with 10% fetal bovine serum (GIBCO-BRL, Scotland, UK) and antibiotics (100 U/mL penicillin and 100 μg/mL streptomycin). The cells were cultured at 37 °C in a humidified atmosphere containing 95% air and 5% CO_2_.

### 4.3. Experimental Protocol

For concentration experiments, AGS cells (2.5 × 10^4/^mL, 5 × 10^5/^10 mL) were treated with lutein (5, 10, or 20 μM) for 20 h (for DNA fragmentation) and 24 h (for cell viability, Bax and Bcl-2 levels, and caspase-3 activation).

For time-course experiments, AGS cells were treated with lutein (20 μM) for 30 min, 1 h, and 2 h (for NADPH oxidase activity) and for 1, 2, 3, 6, and 12 h (for intracellular ROS levels).

For colony formation assay, AGS cells were treated with 20 μM lutein and incubated for 2 weeks, after which the colonies were visualized by staining with crystal violet. Numbers of cell clones were counted under a microscope.

To determine the involvement of NADPH oxidase and ROS, AGS cells were pretreated with NADPH oxidase inhibitor ML171 (20 μM) or antioxidant NAC (1 mM) for 1 h and then treated with lutein (20 μM). Subsequently, cell viability (at 24 h), DNA fragmentation (at 20 h), NF-κB DNA-binding activity, IkBα level, and immunocytochemistry for Nox1 and p47 ^phox^ (at 1 h) were evaluated. To measure the effect of lutein on the viability of normal gastric epithelial cells, RGM-1 cells were treated with lutein (5, 10, or 20 μM) for 24 h.

To investigate the effects of lutein, human gastric cancer MKN-74, MKN-1, and SNU-668 cells were treated with lutein (5, 10, or 20 μM) for 24 h (for cell viability). The cells were treated with or without lutein (20 μM) for 1, 2, and 3 h (for intracellular ROS levels).

To assess whether lutein sensitized gastric cancer cells to the chemotherapeutic drug etoposide, gastric cancer cells (AGS, MKN-74, MKN-1, and SNU-668 cells) and normal RGM cells were used. The cells were pre-treated with lutein (5 μM) for 4 h and then treated with etoposide (10 μM) for 24 h. Cell viability was determined by counting the numbers of viable cells. The concentration of etoposide and pretreatment time with lutein were adapted from the previous study [63].

### 4.4. Preparation of Cell Extracts

The cells were harvested using trypsin-EDTA and pelleted by centrifugation at 1000× *g* for 5 min. The cell pellets were resuspended in lysis buffer containing 10 mM tris (pH 7.4), 1% Nonidet P-40 (NP-40) and commercial protease inhibitor cocktail (complete; Roche, Mannheim, Germany), and lysed by drawing the cells through a 1 mL syringe using several rapid strokes. The mixture was then incubated on ice for 30 min and centrifuged at 13,000× *g* for 15 min. The supernatants were collected and used as whole-cell extracts. To prepare cytosolic and membrane extracts, the cells were extracted in homogenization buffer containing 10 mM tris-HCl (pH 7.4), 50 mM NaCl, 1 mM ethylenediaminetetraacetic acid (EDTA), and a commercial protease inhibitor cocktail, and then centrifuged at 100,000× *g* for 1 h. The pellets were resuspended on ice in lysis buffer containing 50 mM HEPES (pH 7.4), 150 mM NaCl, 1 mM EDTA, and 10% glycerol and were used as membrane extracts. For the isolation of nuclei, cells were extracted in a buffer containing 10 mM HEPES (pH 7.9), 10 mM KCl, 0.1 mM EDTA, 1.5 mM MgCl_2_, 0.05% NP-40, 1 mM dithiothreitol (DTT), and 0.5 mM phenylmethylsulfonylfluoride (PMSF). The nuclear pellets were resuspended on ice in nuclear extraction buffer containing 20 mM HEPES (pH 7.9), 420 mM NaCl, 0.1 mM EDTA, 1.5 mM MgCl_2_, 25% glycerol, 1 mM DTT, and 0.5 mM PMSF, and then centrifuged. The supernatants were used as nuclear extracts.

To prepare the cytosolic and membrane fractions, cells were trypsinized, washed with PBS, and centrifuged at 1000× *g* for 5 min. The cells were then resuspended in lysis buffer containing 10 mM tris, pH 7.4, 50 mM NaCl, 1 mM EDTA, and a commercial protease inhibitor cocktail, then lysed by drawing the cells through a 1 mL syringe using several rapid strokes, and centrifuged at 2000× *g* for 10 min. The supernatant was separated by centrifugation at 100,000× *g* for 1 h. Membrane fractions were obtained by resuspending the pellet in lysis buffer. The supernatants were used as cytosolic fractions. Protein concentration was determined using the Bradford assay (Bio-Rad Laboratories, Hercules, CA, USA).

### 4.5. Measurement of Cell Viability

To measure cell viability, cells were treated with lutein for 24 h and numbers of viable cells were determined by direct counting using a hemocytometer.

### 4.6. Measurement of Intracellular ROS Levels

For the measurement of intracellular ROS, cells were loaded with 10 μM dichlorofluorescein diacetate (DCFH-DA; Sigma-Aldrich) for 30 min. Then, the cells were washed and scraped off using PBS. DCF fluorescence was measured (excitation at 495 nm and emission at 535 nm) using a Victor5 multilabel counter (PerkinElmer Life and Analytical Sciences, Boston, MA, USA).

### 4.7. Measurement of NADPH Oxidase Activity

NADPH oxidase activity was measured by lucigenin assay. The membrane and cytosolic fractions were prepared as described in the section “preparation of cell extracts”. The assay was performed using 50 mM tris-MES buffer (pH 7.0) containing 2 mM KCN, 10 μM lucigenin, and 100 μM NADPH. The reaction was initiated upon the addition of membrane fractions containing 10 μg protein. Photon emission was measured every 60 s for 5 min in a microtiter plate luminometer (Micro-Lumat LB 96V, Berthold, NH, USA). NADPH oxidase activity in the cytosolic extracts was monitored and used as negative control.

### 4.8. Immunocytochemistry

Immunocytochemistry was performed to determine the migration of p47^phox^ to the plasma membrane. The cells were treated with lutein for 1 h or pretreated with ML171 for 1 h before lutein treatment. They were then fixed with 4% formaldehyde. For Nox1 immunostaining, goat pAb (cat. No. ab121009) and anti-goat IgG-R (cat. No. sc-2490) were used. For p47^phox^ immunostaining, mouse pAb (cat. No. sc-17845) and anti-mouse IgG-FITC (cat. No. sc-2099) were used.

### 4.9. Western Blot Analysis

Whole-cell extracts were loaded onto 10–12% SDS-PAGE gels, and proteins were separated by electrophoresis under reducing conditions. Proteins were then transferred onto nitrocellulose membranes by electroblotting. The membranes were blocked using 2–5% non-fat dry milk in tris-buffered saline and 0.2% Tween-20 (TBST) for 1 h at room temperature. They were then incubated with antibodies against actin (sc-1615; Santa Cruz Biotechnology), Bcl-2 (sc-492, Santa Cruz Biotechnology), Bax (sc-526; Santa Cruz Biotechnology), caspase-3 (610323; BD Bioscience, San Jose, CA, USA), cleaved caspase-3 (#9661S; Cell Signaling Technology, Danvers, MA, USA) and IκBα (sc-4094; Santa Cruz Biotechnology) in TBST solution containing 2–5% dry milk, overnight at 4 °C. After washing with TBST, the membranes were incubated with horseradish peroxidase-conjugated anti-mouse or anti-rabbit secondary antibodies in TBST solution containing 2–5% dry milk, for 2 h at 21–23 °C. The proteins were visualized by exposure to an X-ray film using an enhanced chemiluminescence detection system. Actin was used as the loading control. Protein levels of IκBα in whole-cell extracts were compared to that of the loading control actin.

### 4.10. Assessment of DNA Fragmentation

DNA fragmentation was determined by measuring the amount of oligonucleosome-bound DNA in the cell lysate. The relative increase in nucleosomes in the cell lysate, determined at 405 nm, was expressed as an enrichment factor. Nucleosomes were quantified using the sandwich enzyme-linked immunosorbent assay (ELISA) (cell death detection ELISA^PLUS^ kit; cat. No. 11774425001).

### 4.11. Colony Formation Assay

The cells were treated with lutein and incubated for 2 weeks. The cell culture medium was replaced every 2–3 days. When a visible colony appeared, the culture was terminated and the cells were washed twice with PBS. Then, the cells were stained with crystal violet solution for 10 min at 20–25 °C. Colony formation was examined by counting the number of cell clones under the microscope (>50 validated clones).

### 4.12. Electrophoretic Mobility Shift Assay (EMSA)

The shift assay was performed using an NF-κB gel shift oligonucleotide probe (5′-ACTTGAGGGGACTTTCCCAGGGC-3′) obtained from Promega (Madison, WI, USA). An aliquot of the single-stranded oligonucleotides was end-labeled with [^32^P]-deoxyadenosine triphosphate (dATP) (Amersham Biosciences, Piscataway, NJ, USA) using T4 polynucleotide kinase (GIBCO, Grand Island, NY, USA). The radiolabeled oligonucleotides were separated from unincorporated [^32^P] dATP using the Bio-Rad purification column (Bio-Rad Laboratories), and were purified with tris-EDTA buffer. Nuclear extracts isolated from the cells were incubated with [^32^P]-radiolabeled probes in buffer (12% glycerol, 12 mM HEPES (pH 7.9), 1 mM EDTA, 1 mM DTT, 25 mM KCl, 5 mM MgCl2, 0.04 µg/mL poly[d(I-C)]), for 30 min at 21–23 °C. The samples were subjected to electrophoretic separation at 4 °C on a nondenaturing 5% acrylamide gel. The gel was dried at 80 °C for 2 h and exposed to a radiography film at −80 °C, with intensifying screens.

### 4.13. Statistical Analysis

In cases where the experimental group consisted of more than two, one-way analysis of variance followed by Tukey’s post-hoc test was used for statistical analysis. In cases where the experimental group consisted of two, Student’s *t* test was used. All data are presented as mean ± standard error (the total number of samples in each group was 12). A *p* value less than 0.05 was considered statistically significant.

## Data Availability

The data used to support the findings of this study are included in this article.

## Abbreviations

dATP	Deoxyadenosine triphosphate
CAD	Caspase-activated DNase
DCF	Dichlorofluorescein
DCFH-DA	Dichlorofluorescein diacetate
DMEM	Dulbecco’s modified Eagle’s medium
DMSO	Dimethyl sulfoxide
DTT	Dithiothreitol
EDTA	Ethylene diaminetetraacetic acid
ELISA	Enzyme-linked immunosorbent assay
EMSA	Electrophoretic mobility shift assay
ERK	Extracellular signal-regulated kinase
FITC	Fluorescence-activated single cell sorting
HEPES	Hydroxyethyl piperazine ethane sulfonicacid
ICAD	Inhibitor of caspase-activated DNase
MAPK	Mitogen-activated protein kinase
NAC	N-acetycysteine
NADPH	Reduced nicotinamide adenine dinucleotide phosphate
NF-κB	Nuclerar factor-κB
NP-40	Nonidet P-40
Nrf2	Nuclear factor erythroid 2–related factor 2
PARP	Poly (ADP-ribose) polymerase
PMSF	Phenylmethylsulfonylfluoride
ROS	Reactive oxygen species
TBST	Tris-buffered saline and 0.2% Tween-20
TRAIL	TNF-related apoptosis-inducing ligand
